# Dry Knee Arthroscopy With Carbon Dioxide Insufflation for Anterior Cruciate Ligament Reconstruction

**DOI:** 10.1016/j.eats.2024.102986

**Published:** 2024-03-24

**Authors:** Umer Butt, Zainab Aqeel Khan, Muhammad Ali Ahmad Sheikh, Anders Stålman, Filip Vuletić

**Affiliations:** a Department of Trauma and Orthopaedic, AO Hospital, Karachi, Pakistan; b Sulis Hospital Bath, Bath, U.K.; c Kantonsspital Baselland, Liestal, Switzerland; d Stockholm Sports Trauma Research Center, Department of Molecular Medicine and Surgery, Karolinska Institutet, Stockholm, Sweden; e Capio Artro Clinic Sophiahemmet Private Hospital, Stockholm, Sweden; f Department for Orthopaedic and Trauma Surgery, University Hospital “Sveti Duh,” Sveti Duh, Zagreb, Croatia; g Faculty of Kinesiology, University of Zagreb, Zagreb, Croatia

## Abstract

Anterior cruciate ligament (ACL) injuries are commonly treated through orthopaedic surgery, with traditional procedures relying on arthroscopy using fluid as the medium. However, dry arthroscopy has emerged as a potentially advantageous alternative technique. This method allows the knee joint to remain dry, reducing the risk of fluid leakage and enabling a more precise surgical visualization, resulting in shorter operation times and fewer complications. Recent research has highlighted the benefits of carbon dioxide (CO_2_) insufflation during ACL reconstruction, which can decrease pain and discomfort during early recovery. This article introduces a technique for performing ACL reconstruction that eliminates the need for arthroscopic fluid for visualization or instrumentation. Based on CO_2_ insufflation, this technique shows promise as a viable alternative to traditional fluid distention methods.

The anterior cruciate ligament (ACL) is the most commonly injured ligament that requires reconstruction in orthopaedic surgery.^1^ Orthopaedic surgeons always look for ways to improve surgical techniques to enhance patient outcomes and optimize treatment strategies. Although ACL reconstruction procedures have significantly evolved in recent decades, fluid has remained the unchanged gold standard as a medium for arthroscopy and as a mechanism for visualizing and instrumenting the joint. However, since the 1980s, several authors have recognized that dry arthroscopy may be superior to fluid arthroscopy.^2^^,^^3^ Dry knee arthroscopy is a specialized procedure where the knee joint is accessed using specific instruments while maintaining a dry environment. Unlike conventional arthroscopy, where fluid is used for joint distention and visualization, dry knee arthroscopy relies on targeted joint distraction with gas insufflation and advanced imaging techniques to better visualize intra-articular structures.^2^^,^^3^ The technique that eliminates the need for fluid joint distension may have several potential benefits. First, it may offer improved visualization during the surgical procedure, giving surgeons a better view of the surgical site.^3^ Second, it may enhance surgical precision, particularly in accurately measuring and placing the femoral tunnel.^2^^,^^4^ Third, it has been shown to reduce the time required for tourniquet application.^2^ These advantages can potentially lead to better postoperative recovery and higher patient satisfaction.^5^ In addition, the “dry” visual quality facilitates static and dynamic assessment of the physiology of the knee joint and its potential structural and biomechanical pathology.^4^^,^^6^ Despite the subjective appreciation of dry arthroscopy, the theoretical risk of gas embolism during CO_2_ knee insufflation makes it essential to study its safety objectively. Imbert and Schlatterer^2^ conducted a study investigating the risk of potential systemic complications by monitoring end-tidal carbon dioxide concentration. Their study showed no increased risk of hematogenous gas leak, and the measured end-tidal carbon dioxide concentrations were similar to those measured during fluid arthroscopy.^2^

In this article, we explain the technique of dry knee arthroscopy with CO_2_ insufflation for ACL reconstruction while considering the existing literature and clinical studies. We discuss this approach’s potential benefits and challenges and present the evidence supporting its use. We aim to contribute to the growing body of knowledge on improving techniques for ACL reconstruction by examining the existing evidence and incorporating our experience.

## Surgical Technique

In this article, we present our technique for performing dry knee arthroscopy with CO2 insufflation (Video 1). The steps involved in our surgical technique, along with important tips and potential challenges, are detailed in Table 1.

**Table 1:** Surgical Steps, Pearls, and Pitfalls of Dry Knee Arthroscopy With Carbon Dioxide Insufflation in ACL Reconstruction

Surgical Step	Pearls	Pitfalls
Anteromedial portal preparation	Introduction of a hemostat clamp or a blunt trocar to debride the soft tissue and puncture the joint capsule to provide a sealing effect of soft tissue and help reduce gas leakage	An inappropriate anteromedial portal can cause additional gas leakage and problems with joint distension.
Placement of the Passport Button Cannula and Hoffa fat pad debridement	It helps reduce Hoffa fat pad view obstruction and helps visualize the intercondylar notch.	Not placing the cannula obscures the vision of the intercondylar notch with Hoffa fat pad interposition.
Graft preparation	Little muscle tissue is left on the tendon to potentially promote graft healing and aid in graft thickness.	
Drilling of tibial ACL tunnel	The use of a 55° tibial guide allows adequate tunnel length. The target of the tibial guide is placed onto the ACL remnant.	

ACL, anterior cruciate ligament.

### Patient Setup

**Fig 1:**
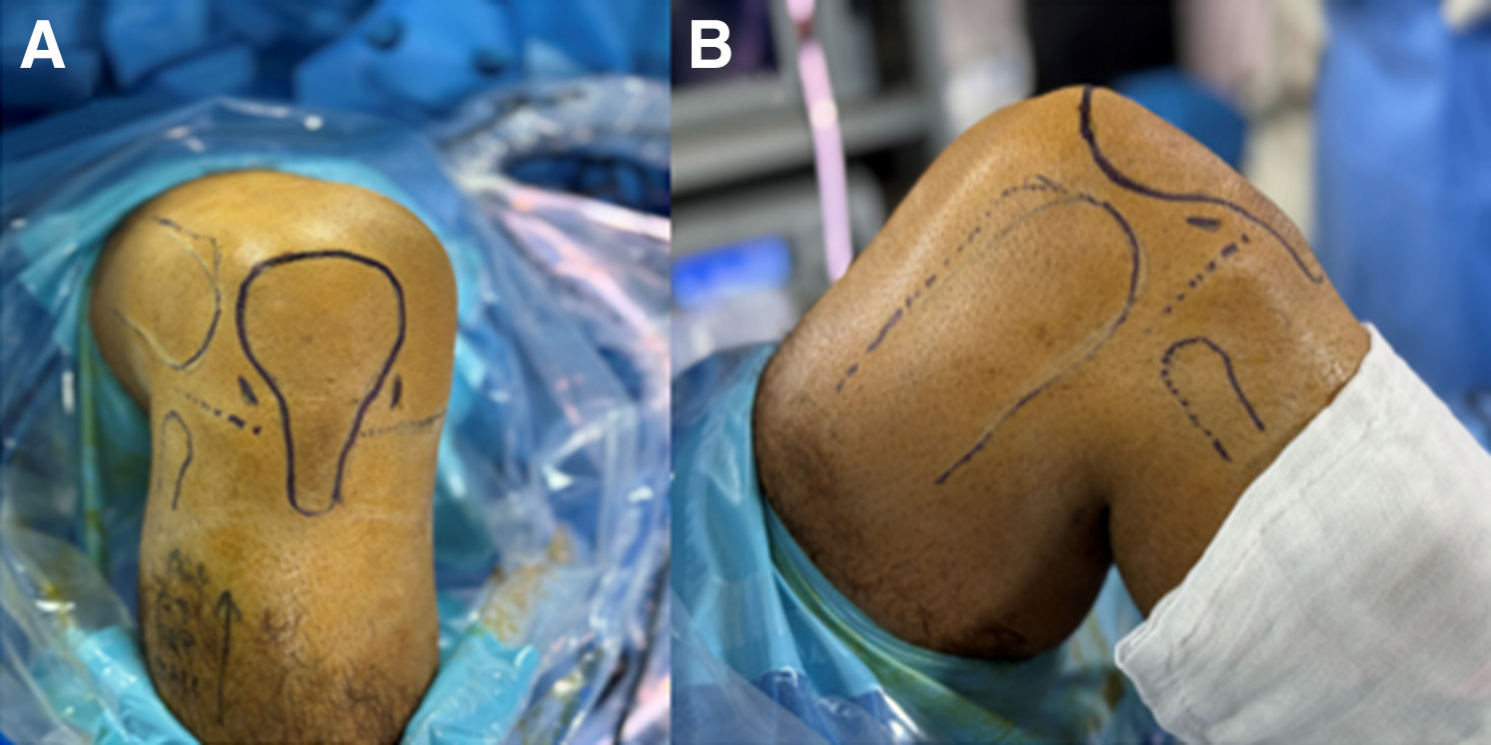
The patient positioned supine with a standard lateral post proximal to the right knee. A tourniquet is applied around the thigh, the limb is prepared and draped in the usual manner, and anatomic landmarks are drawn. (A) View from the anterior side. (B) View from the lateral side.

**Fig 2:**
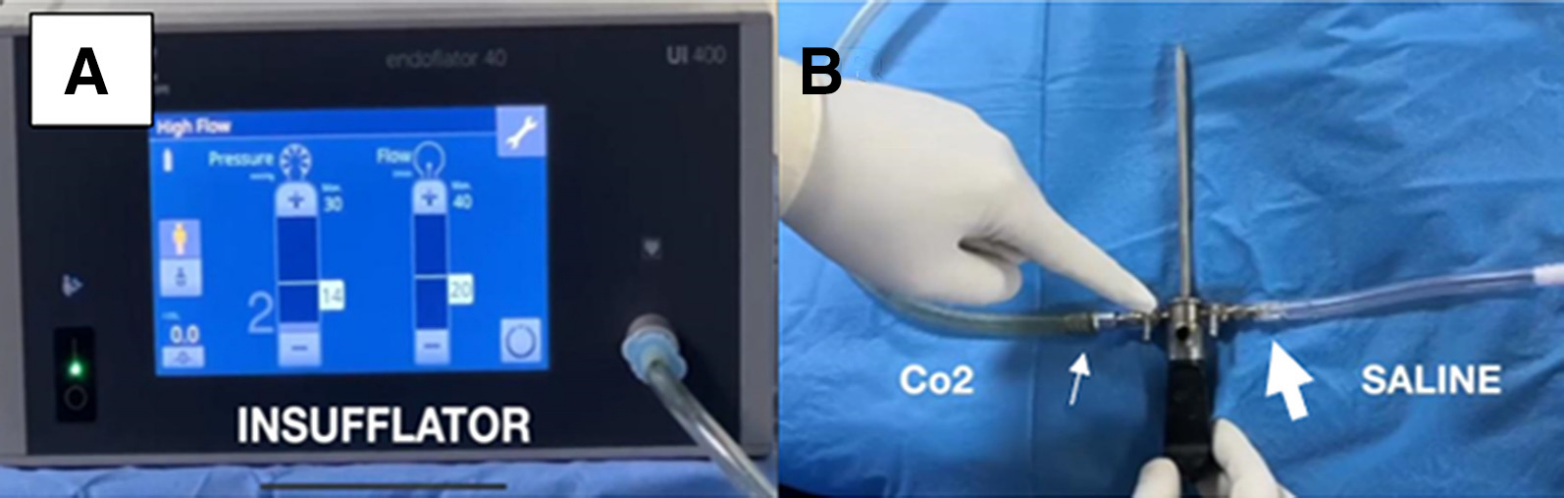
(A) Modified laparoscopic insufflator. (B) First entry side (small arrow) of the arthroscope is connected with an insufflator to fill carbon dioxide gas. Normal saline is connected to the arthroscope’s second entry side (large arrow). (CO_2_, carbon dioxide.)

**Fig 3:**
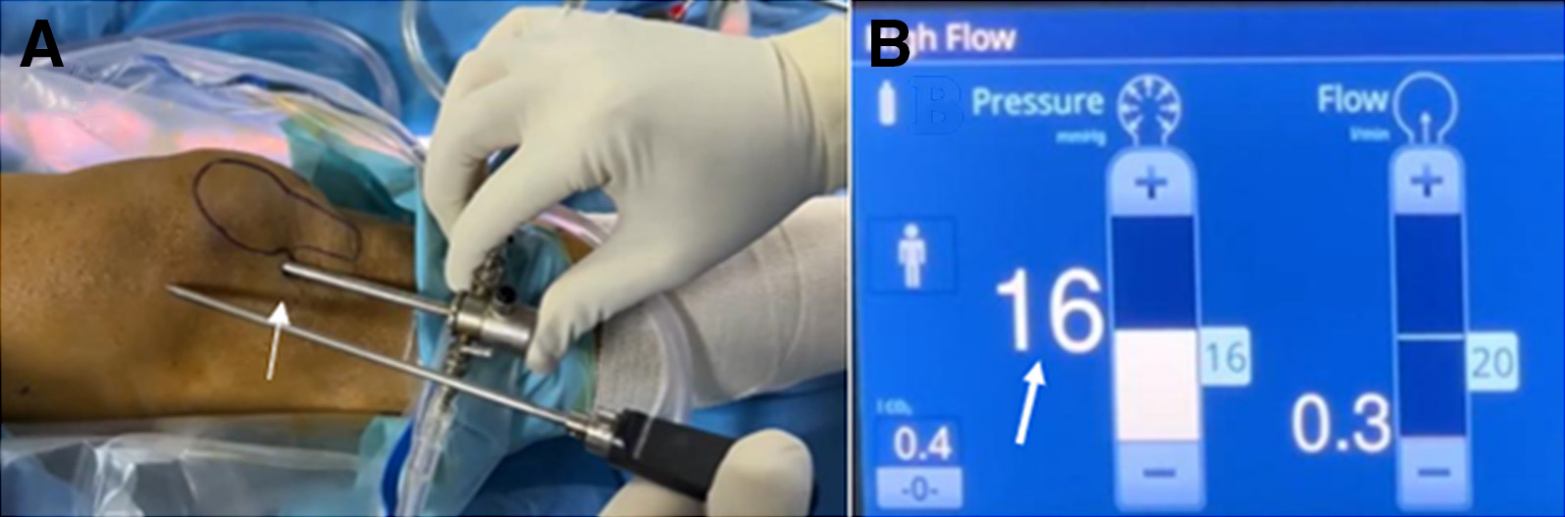
(A) Arthroscope placed in a high anterolateral portal of right knee (arrow). (B) A modified laparoscopic insufflator fills carbon dioxide gas within the knee joint up to a pressure of 20 mm Hg (arrow).

The patient is positioned supine, with a standard lateral post proximal to the knee, at the level of a padded tourniquet and leg support to keep the knee free throughout the range of motion (ROM) (Fig 1). A tourniquet is applied around the thigh, and the limb is prepared and draped as usual. The lower extremity is drained of blood with an Esmarch bandage, and the tourniquet is inflated to 300 mm Hg. After a high anterolateral approach, a modified laparoscopic insufflator connected to an arthroscope is used to fill CO_2_ gas within the knee joint up to a pressure of 20 mm Hg (Figs 2 and 3). The position of the standard anteromedial approach is created under direct vision, first assessed with a needle, and then with the 11-point scalpel, skin is incised. Following, a hemostat clamp or a blunt trocar is introduced to debride the soft tissue and puncture the joint capsule to provide a sealing effect of soft tissue. A dry diagnostic arthroscopy is performed, and the Passport Button Cannula (Arthrex) is placed in an anteromedial portal. Normal saline is connected to the second entry side of an arthroscope to clear any residual synovial fluid or hemarthrosis and then aspirated using a suction pump placed on a shaver. If any remaining Hoffa fat pad obstructs clear vision after joint insufflation, it is removed by a shaver.

### Graft Harvest and Preparation

The semitendinosus and gracilis tendons are harvested through a vertical incision located 1 cm medial to the tibial tuberosity using a closed-end tendon stripper (Arthrex). The tendons are sutured at the proximal and distal ends with a whipstitch and then bent over the TightRope (Arthrex) on the femoral side, ensuring a graft width of 8 to 8.5 cm. To secure further manipulation and tension during fixation, a No. 2 FiberWire (Arthrex) is attached to the tibial end of the graft.

### Femoral Tunnel Placement and Drilling

**Fig 4:**
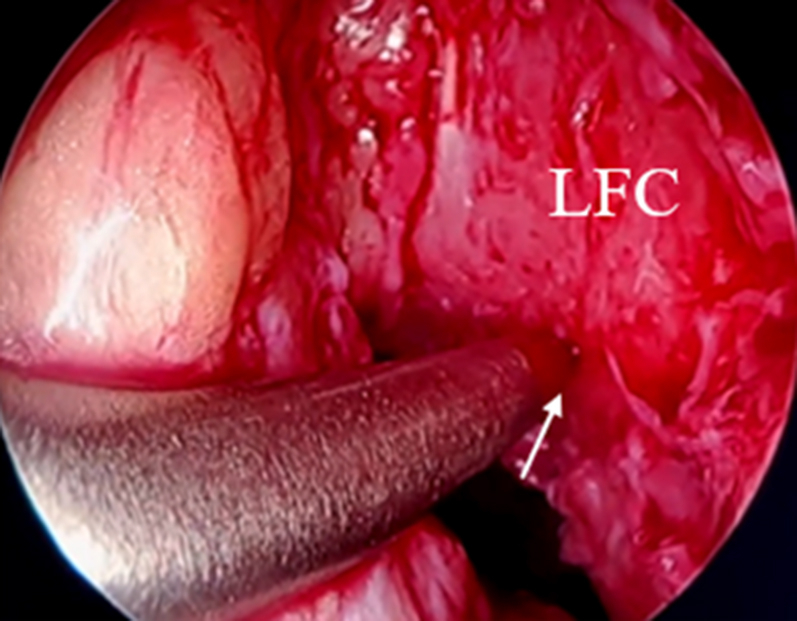
An awl is inserted through the anteromedial portal, and a mark is made on the anatomic femoral footprint of the anterior cruciate ligament (arrow) (right knee, anterolateral portal view)—a clear view of the medial wall of the lateral femoral condyle. (LFC, lateral femoral condyle.)

**Fig 5:**
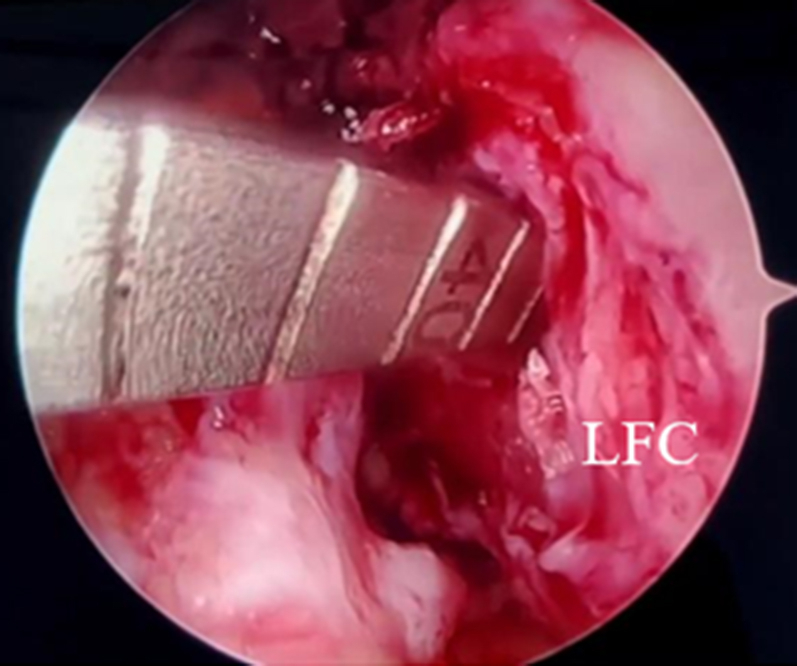
A measuring passing pin to ensure tunnel sizing accuracy (right knee, anterolateral portal view). (LFC, lateral femoral condyle.)

When a clear view of the notch is made and the medial wall is prepared, an awl is inserted through the anteromedial portal, and a mark is made on the anatomic femoral footprint of the ACL (Fig 4). First, a 4.5-mm drill bit is used to drill through the femur. A measuring 4.5-mm drill (Arthrex) and a passing pin ensure tunnel sizing accuracy (Fig 5). Next, using the inside-out technique, the femoral bone is prepared to the appropriate size for the ACL. The proximal portion of the femoral bone tunnel is cleaned with a shaver to reduce soft tissue entrapment. Any residual bone or soft tissue is irrigated and removed with an irrigation saline.

### Tibial Tunnel Placement and Drilling

**Fig 6:**
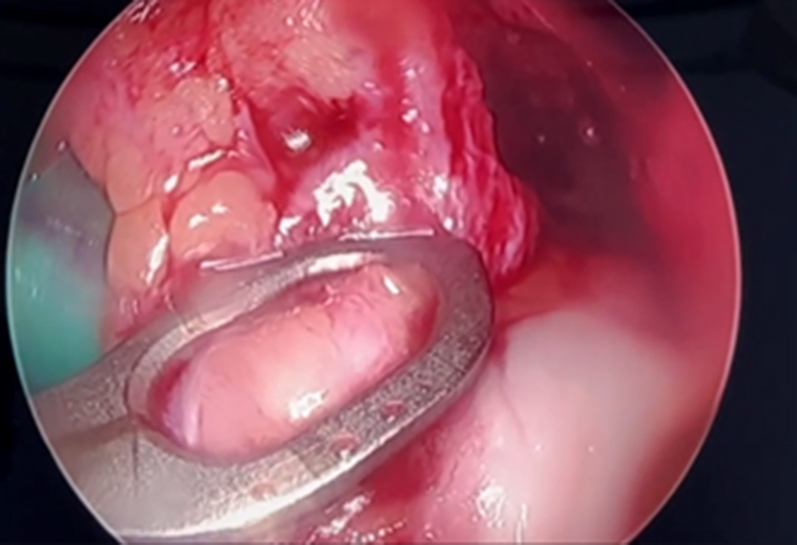
Arthroscopic view of the right knee through the anterolateral portal with the tibial guide targeting the center of the anterior cruciate ligament stump.

**Fig 7:**
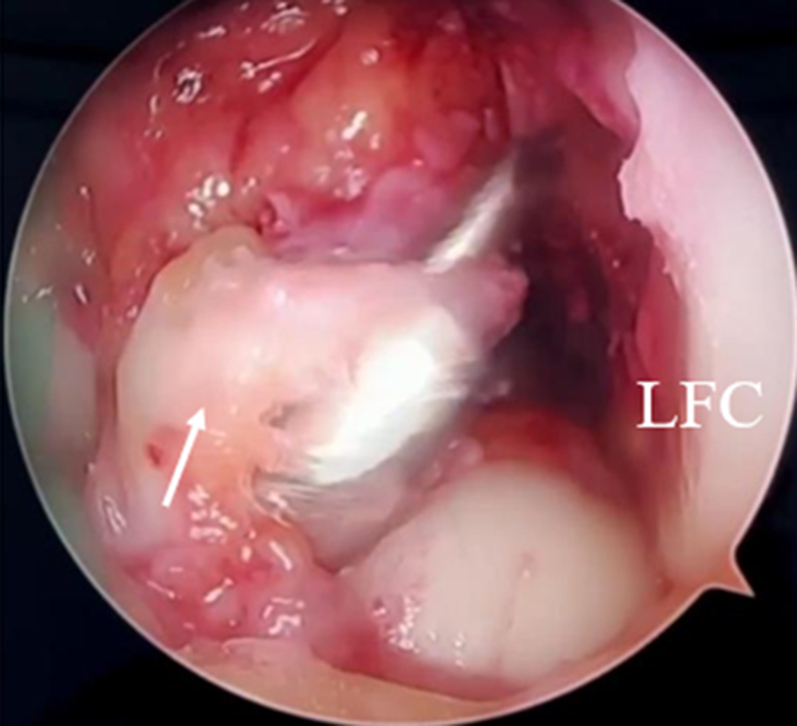
Outside-in reaming using the cannulated drill set is performed according to the previously measured size of the anterior cruciate ligament (ACL) graft while preserving the synovial covering of the ACL remnant (arrow) and the tibial attachment (right knee, anterolateral portal view). (LFC, lateral femoral condyle.)

The tibial guide is placed at a 55° angle, targeting the center of the ACL stump (Fig 6). Subsequent outside-in reaming using the cannulated drill set is performed according to the previously measured size of the ACL graft while preserving the synovial covering of the ACL remnant and the tibial attachment (Fig 7).

### Graft Passage

**Fig 8:**
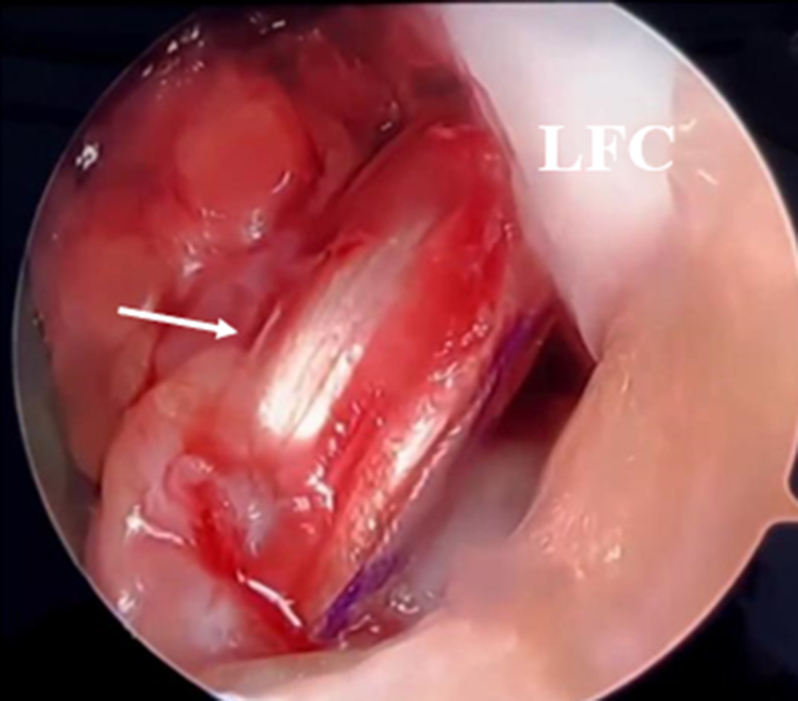
Anterolateral portal view of the right knee showing the anterior cruciate ligament graft (arrow). (LFC, lateral femoral condyle.)

The TightRope and graft are passed through the tibial and femoral tunnels using a passing suture. The TightRope is then tightened until the proximal 2 cm of the graft reaches the inside of the femoral tunnel (Fig 8). The knee is then placed in 90° of flexion, and the ACL graft is tightened and secured with a FastThread (Arthrex) bioresorbable interference screw whose width and length are determined according to the size of the ACL graft and the length of the tibial tunnel. Graft tension is checked by performing an ROM test from 90° to 0° of flexion. The TightRope is additionally tightened on the femoral side to apply additional tension if necessary.

### Postoperative Course

Immediate full weightbearing without a brace, 0° to 90° ROM, and closed-chain strengthening exercises are allowed. Early rehabilitation focuses on achieving full extension and activation of the medial vastus. Progressive full ROM exercises are started after the first 4 weeks after surgery.

## Discussion

In our experience, gas insufflation in arthroscopic ACL reconstruction may have several potential advantages and some possible risks and disadvantages, as outlined in Table 2. Similar to previous research, dry arthroscopy provides excellent visualization, allowing for the accurate identification of intra-articular structures and potentially reducing the risk of technical errors.^2^^,^^4^ It results in less bleeding during surgery, providing clearer visualization of anatomic landmarks, allowing surgeons to place tunnels more accurately.^2^^,^^4^ Improved precision can potentially improve graft positioning, ultimately leading to better clinical outcomes. Another potential benefit is less risk of fluid extravasation into soft tissue. This finding is consistent with previous studies showing reduced swelling and improved early recovery after dry arthroscopic procedures for various joint surgeries.^2^^,^^3^^,^^7^^,^^8^ Reduced swelling may alleviate postoperative pain, improve early mobilization, and accelerate rehabilitation.^5^ While our study demonstrates a promising technique, it is essential to acknowledge its limitations and consider the broader literature when interpreting the findings. It is essential to consider the drawbacks of using this dry arthroscopy technique. This method requires installing a system for both fluid irrigation and gas insufflation. During surgery, alternating rinsing and working phases are necessary, and the joint cavity cannot be distended as effectively with gas as it is with fluid. Additionally, this technique is not suitable if radiofrequency is desired. In our opinion, the only potential risk associated with this technique is the development of local subcutaneous emphysema. As mentioned, the literature suggests no increased systemic risk of a hematogenous gas leak. The current research on dry arthroscopy for ACL reconstruction is still relatively limited, with only a few studies specifically addressing this technique. However, the existing literature on dry arthroscopy in other joint surgeries supports the potential benefits observed in our study.^2^^,^^4^^,^^7^ Long-term follow-up with a control group is necessary to evaluate the durability and effectiveness of dry arthroscopy-based ACL reconstruction.

**Table 2:** Potential Advantages, Disadvantages, and Risks of Dry Anterior Cruciate Ligament Reconstruction

Advantages	Disadvantages and Risks
More anatomic, “nonaquarium” vision	Development of local subcutaneous emphysema
Better view of the intercondylar notch^4^	Addition of “air”/CO_2_ system
More precise placement and measurement of the femoral tunnel^4^	Joint space/distension depends on CO_2_ pressure
Prevents portal shifting/soft tissue layer obliterations	Inadequate if radiofrequency is desired
Less intra-/postoperative fluid extravasation	Occasional debris fluid washout is needed

CO_2_, carbon dioxide.

In conclusion, our study adds to the increasing body of research on using dry arthroscopy in ACL reconstruction. Our experience suggests that dry arthroscopy can provide good visualization and the possibility of excellent surgical precision as a prerequisite for good postoperative outcomes. However, few clinical studies have been performed, and studies with larger sample sizes, a control group, and more long-term follow-up are necessary to verify our findings and assess their clinical relevance.

## Disclosures

The authors declare that they have no known competing financial interests or personal relationships that could have appeared to influence the work reported in this paper.

## Supplementary Data

ICMJE author disclosure forms

ICMJE author disclosure forms

ICMJE author disclosure forms

ICMJE author disclosure forms

ICMJE author disclosure forms

Video 1 This video explains our dry knee arthroscopy technique using intermittent carbon dioxide gas insufflation for anterior cruciate ligament (ACL) reconstruction. The patient is supine with a standard lateral post proximal to the knee. A tourniquet is applied around the thigh, and the limb is prepared and draped in the usual manner. Anatomic landmarks are drawn, and the lower extremity is drained of blood with an Esmarch bandage. The tourniquet is then inflated to 300 mm Hg. A modified laparoscopic insufflator is connected to an arthroscope. Normal saline is connected to the arthroscope’s second entry side, to clear any synovial fluid or hemarthrosis, which is then aspirated using a suction pump attached to a shaver. After placing a standard anterolateral portal, an insufflator fills the knee joint with carbon dioxide gas up to a pressure of 20 mm Hg. During a dry diagnostic arthroscopy, the surgeon creates the position for the standard anteromedial approach under direct vision. This is first assessed with a needle and then with an 11-point scalpel, after which the skin is incised. Following this, a hemostat clamp or a blunt trocar is used to remove any soft tissue and puncture the joint capsule, which helps to provide a sealing effect of soft tissue. This creates an anteromedial portal for the Passport Button Cannula. If any remaining Hoffa fat pad obstructs clear vision after joint insufflation, it is removed by a shaver. When a clear view of the notch is made and the medial wall is prepared, an awl is inserted through the anteromedial portal, and a mark is made on the anatomic femoral footprint of the ACL. The semitendinosus and gracilis tendons are harvested through a vertical incision located 1 cm medial to the tibial tuberosity using a closed-end tendon stripper. The tendons are sutured at the distal end with a whipstitch and then bent over the TightRope on the femoral side, ensuring a graft width of 8 to 8.5 cm. To secure further manipulation and tension during fixation, a No. 2 FiberWire is attached to the tibial end of the graft. First, a 4.5-mm drill bit is used to drill through the femur. A measuring guide and passing pin are used to ensure accuracy. Next, using the inside-out technique, the femoral bone is drilled to the appropriate size for the ACL. After drilling, the proximal portion of the femoral bone tunnel is cleaned with a shaver to reduce the risk of soft tissue entrapment. Finally, any remaining bone or soft tissue is irrigated and removed using saline. The tibial guide is positioned at a 55° angle to target the center of the ACL stump. The next step involves using the cannulated drill set to ream the bone from the outside-in based on the previously measured ACL graft size. The synovial covering of the ACL remnant and the tibial attachment are carefully preserved throughout the procedure. The TightRope and graft are passed through the tibial and femoral tunnels using a passing suture. The TightRope is then tightened until the proximal 2-cm mark of the graft reaches approximately the inside of the femoral tunnel. The knee is then placed in 90° of flexion, and the ACL graft is tightened and secured with a bioresorbable interference screw whose width and length are determined according to the size of the ACL graft and the length of the tibial tunnel. Graft tension is checked by performing a range of motion test from 90° to 0° of flexion. If necessary, the TightRope system is used to apply additional tension. Upon completion of the procedure, a thorough examination of the tissue indicates no noticeable swelling in the knee area.
